# Supplementary figures and images for: DDX6 modulates P-body and stress granule assembly, composition, and docking
Source: J Cell Biol. 2024 Mar 27;223(6):e202306022. doi: 10.1083/jcb.202306022 (PMC10978804; doi:10.1083/jcb.202306022)

Source data for Fig. S2 b

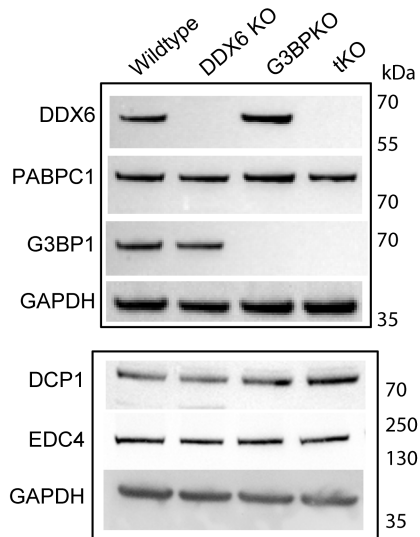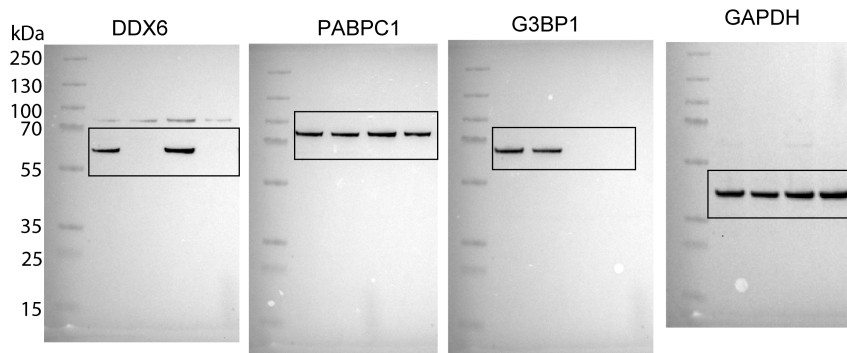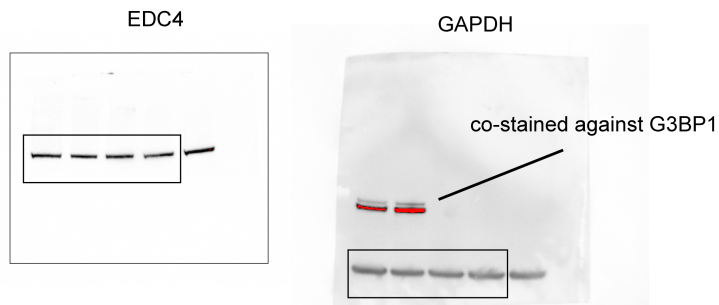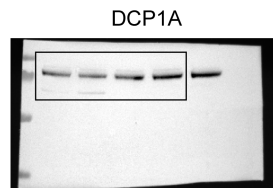

Supplement: SourceData FS2 — is the source file for Fig. S2. [file JCB_202306022_SourceDataFS2.pdf]

Source data for Fig. S4 a

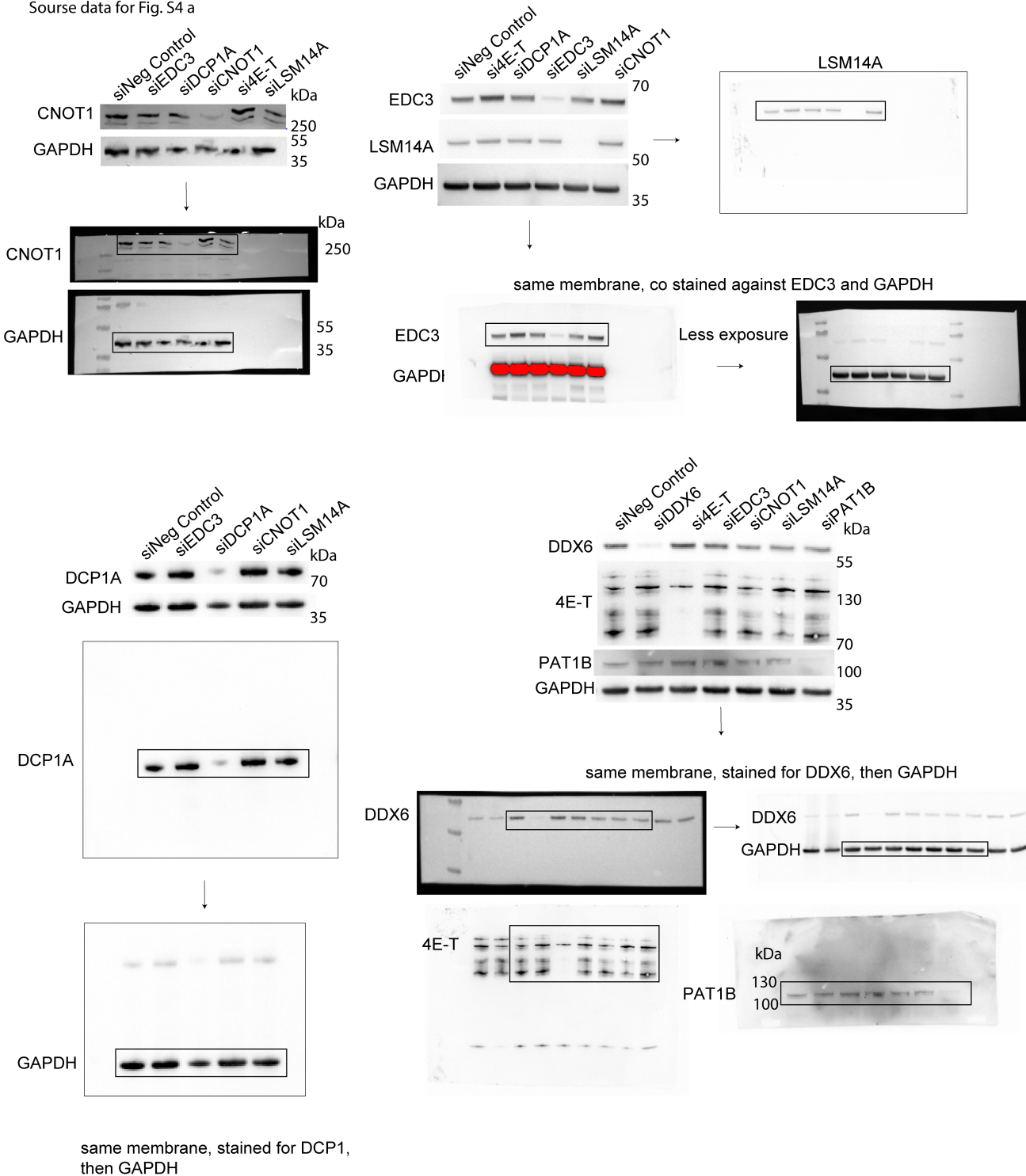

Supplement: SourceData FS4 — is the source file for Fig. S4. [file JCB_202306022_SourceDataFS4.pdf]

Source data for Fig. S5c

DDX6 KO cells

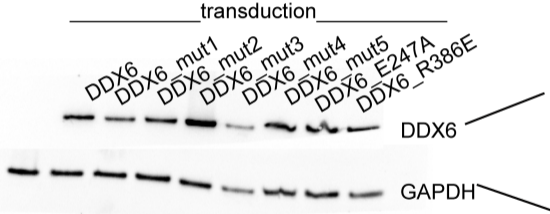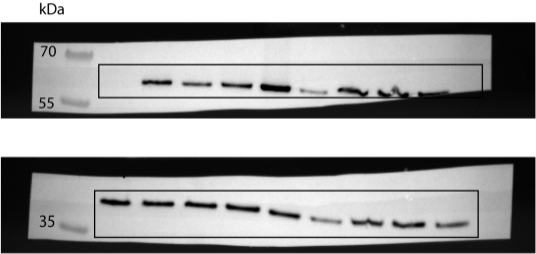

Supplement: SourceData FS5 — is the source file for Fig. S5. [file JCB_202306022_SourceDataFS5.pdf]
